# Ubiquity and Diversity of Cold Adapted Denitrifying Bacteria Isolated From Diverse Antarctic Ecosystems

**DOI:** 10.3389/fmicb.2022.827228

**Published:** 2022-07-18

**Authors:** Angela Cabezas, Gastón Azziz, Patricia Bovio-Winkler, Laura Fuentes, Lucía Braga, Jorge Wenzel, Silvia Sabaris, Silvana Tarlera, Claudia Etchebehere

**Affiliations:** ^1^Instituto Tecnológico Regional Centro Sur, Universidad Tecnológica, Durazno, Uruguay; ^2^Laboratorio de Microbiología, Departamento de biología, Facultad de Agronomía, UdelaR, Montevideo, Uruguay; ^3^Laboratorio de Ecología Microbiana, Departamento de Bioquímica y Genómica Microbiana, Instituto de Investigaciones Biológicas Clemente Estable, Montevideo, Uruguay; ^4^Laboratorio de Ecología Microbiana Medioambiental, Departamento Biociencias, Facultad de Química, Montevideo, Uruguay

**Keywords:** denitrification, isolation, Antarctica, denitrifying bacteria, nitrogen cycle, cold environments

## Abstract

Nitrogen cycle has been poorly investigated in Antarctic ecosystems. In particular, how extreme conditions of low temperature, dryness, and high radiation select the microorganisms involved in the cycle is not yet understood. Denitrification is an important step in the nitrogen cycle in which nitrate is reduced stepwise to the gases NO, N_2_O, and N_2_. Denitrification is carried out by a wide group of microorganisms spread in the phylogenetic tree. The aim of this work was to isolate and characterize denitrifying bacteria present in different cold environments from Antarctica. Bacterial isolates were obtained from lake, meltwater, sea, glacier ice, ornithogenic soil, and penguin feces samples from King George Island, Fildes peninsula in the Antarctic. Samples were taken during the deicing season in five sampling campaigns. From all the samples we were able to isolate denitrifying strains. A total of 199 bacterial isolates with the capacity to grow in anaerobic mineral media reducing nitrate at 4°C were obtained. The characterization of the isolates by 16S rRNA gene sequence analysis showed a high predominance of the genus *Pseudomonas*, followed by *Janthinobacterium*, *Flavobacterium*, *Psychrobacter*, and *Yersinia*. Other minor genera detected were *Cryobacterium*, *Iodobacter*, *Kaistella*, and *Carnobacterium*. The capacity to denitrify was not previously described for most of the bacteria related to our isolates and in many of them denitrifying genes were not present suggesting the presence of new genes in this extreme environment. Our work demonstrates the ubiquity of denitrification in the Maritime Antarctica and gives important information linking denitrification at cold temperature with taxa in an unequivocal way.

## Introduction

Denitrification is an anaerobic respiration in which nitrate, nitrite, nitric, and nitrous oxides are used as terminal electron acceptors. It is performed by a diverse and polyphyletic group of microorganisms that includes species from the three domains of life (Zumft, 1997). For most denitrifiers, denitrification is an ancillary respiration, activated in response to oxygen depletion and the presence of NO_3_^−^, NO_2_^−^, or N_2_O (Hayatsu et al., 2008). However, some microorganisms are aerobic denitrifiers and perform denitrification under aerobic conditions (Yang et al., 2020). This implies that denitrifiers inhabit many environments and are not restricted to the conditions that are conducive to denitrification. The process is carried out in a stepwise fashion that involves the sequential reduction of the molecules involved and when the pathway is complete, molecular nitrogen is formed. However, not all denitrifiers have the genes that code for the complete pathway. For instance, some denitrifiers only perform the first steps, while others only include the nitrous oxide reductase gene in their gene repertory (Jones et al., 2014). Even more, the regulation of the expression of these genes is modular which results in the differential activation of the steps. This can result in different ratios of N_2_O/N_2_ produced by denitrification, which depends on the denitrifying communities present and on the environmental conditions that regulate the process (Ai et al., 2017). As a process dependent on enzymatic reactions, denitrification is usually considered to be boosted by increasing temperatures, as long as it does not compromise the functioning of the cells involved (Tan et al., 2020). Nevertheless, denitrifying microorganisms have been isolated from extreme environments and shown to be able to denitrify well beyond the limits of the optimal temperature range for denitrification (Torregrosa-Crespo et al., 2018). Moreover, there is clear evidence that denitrification is an important process at low temperatures (Cavaliere and Baulch, 2018).

The Antarctic continent, and the different geographical regions that compose it, represent a unique environment. It is characterized by an extreme cold and arid climate, intense UV radiation, and minimal direct anthropogenic influence (López-Martínez, 2020). King George Island is located in the Maritime Antarctica, where climatic conditions are milder than in the Continental Antarctica. In Maritime Antarctica, the climate has been changing rapidly since last century, with the mean annual temperature showing an increase of at least 3.4°C over the past 50 years (Bockheim et al., 2013). This warming has caused a decrease of the permafrost layer and glaciers retreat, resulting in a more humid environment. Surface liquid freshwater can be found in lakes and streams which are formed during summer as the result of ice melting (Callejas et al., 2018). Also, the presence of fauna such as birds and mammals results in organic matter input, which in the case of penguins determine the formation of ornithogenic soils (Tatur and Myrcha, 1984). Interestingly, Antarctica also represents a potential reservoir of endemic microbial species (Vyverman et al., 2010). Denitrification in the Antarctic environment has been less studied compared to other environments on Earth, even when compared to other cold environments (Palacin-Lizarbe et al., 2018). However, the understanding of denitrification in this region of the Earth would be particularly valuable. Nitrous oxide, one of the products of denitrification, is a greenhouse gas with a potential of more than 300 times that of CO_2_. Besides, it is an ozone layer depleting gas (Jones et al., 2014), an environmental issue that is critical in Antarctica (Abbasi and Abbasi, 2017).

The development of molecular techniques, such as next generation sequencing, and its application on the study of microbial populations has allowed a deeper understanding of the ecology of microorganisms in its natural environment (Mocali and Benedetti, 2010). However, for denitrifying bacteria, horizontal gene transfer events of denitrification genes make it difficult to construct denitrification-based phylogenies (Heylen et al., 2006). Moreover, primer coverage issues suggest that many organisms capable of denitrification are not identified in environmental surveys (Green et al., 2010). Culture-based approaches, with obvious limitations, improves the detection of environmentally relevant taxa and allows a better understanding of the physiology of psychrophilic denitrifying bacteria, linking functions to taxa in an unequivocal way (Paul et al., 2018). Moreover, the isolation of bacteria allows to recover genomes to incorporate into the sequence databases.

The main objective of this work was to identify the main cultivable denitrifiers in different Antarctic ecosystems. Diversity of denitrifying bacteria is scarcely known in Antarctica. In particular, few studies focus on isolating denitrifying strains. In this work, potential denitrifying bacteria were isolated from samples taken from lakes (water and sediments), meltwater, sea (water and sediments), ice from a glacier, ornithogenic soil, and penguin feces in different points of King George Island. The samples were taken at different times during the deicing period. The isolates were characterized by 16S rRNA gene sequencing and their denitrifying potential activity was tested at 4°C. A bioinformatic approach was used to identify possible genes involved in the denitrification process.

## Materials and Methods

### Site Description and Sampling

**Figure 1:**
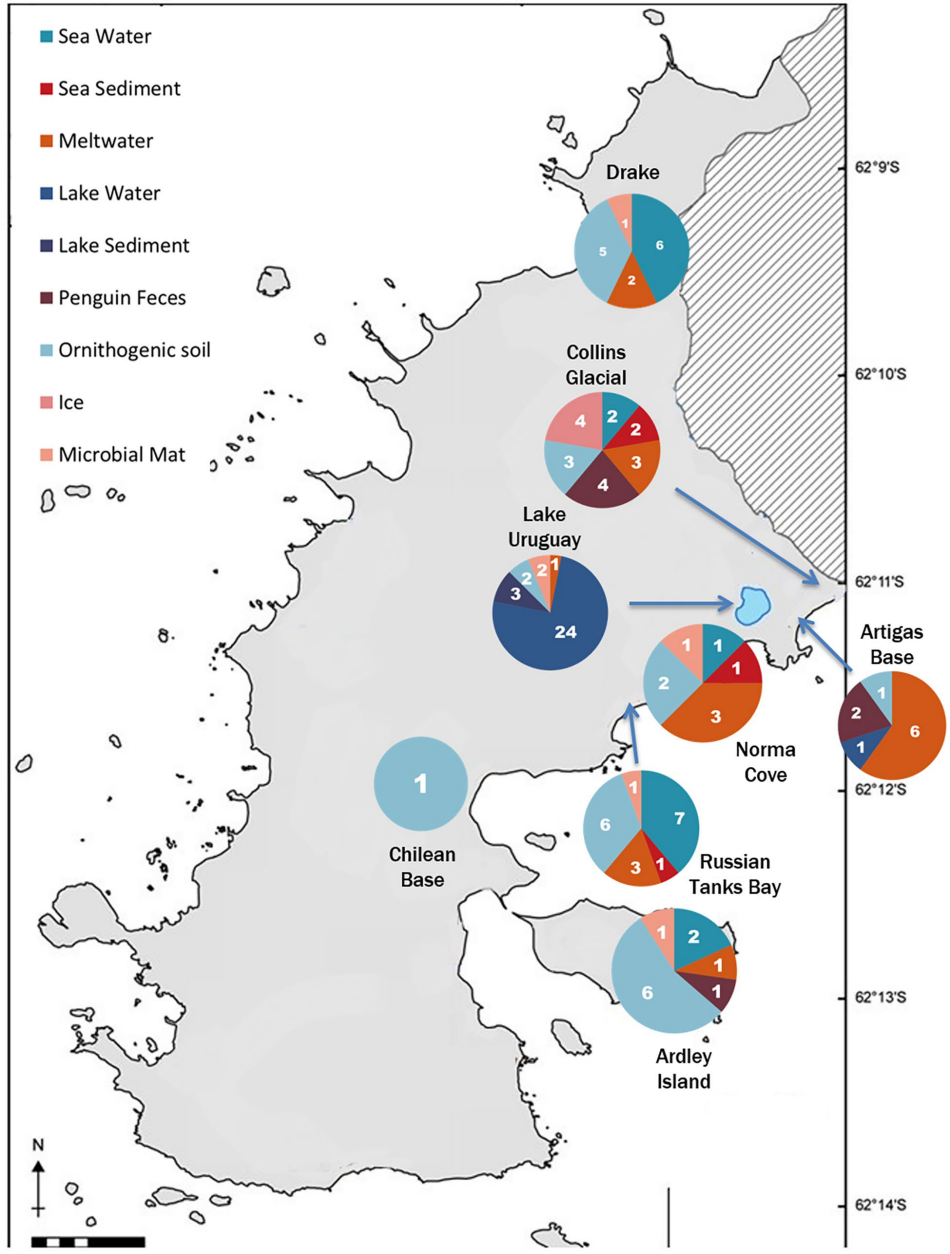
Map of the Fildes peninsula on the King George Island showing sampling points. The number of samples collected from each location are shown according to sample type.

The study was carried out in the vicinity of the José Artigas Antarctic Base (62°11′04′S 58°51′07′W), which is located on King George Island South Shetlands Archipelago, Maritime Antarctica, 100 km from the Antarctic Peninsula. Samples were taken during five sampling campaigns (March 2012, December 2012, January 2013, March 2013, and December 2013) as indicated in Table 1 during deicing seasons (December is the beginning of the deicing and March is the end of the summer season). Samples from lakes (water and sediments), meltwater streams, microbial mats, sea water and sediments, ice from a glacier, ornithogenic soil, and penguin feces were taken from different locations (Figure 1). Approximately 10 g were collected for solid samples. For liquid samples, approximately 1 L was collected in the first sampling campaign (March 2012) and 250 ml for the following campaigns. The samples were collected using sterile containers and stored on ice until processing. In the first campaign (March 2012), an exhaustive sampling of the lakes was performed. Three different points of the lakes were sampled at different heights of the water column. However, no difference regarding the diversity of denitrifying bacteria was observed in the different lake samples. Therefore, in the following campaigns, less lake samples were collected (Supplementary Table S1). Moreover, in the campaigns performed in December 2012 and 2013, most of the lake-water was still frozen, and samples were collected from the water pipe that supplies water to the Artigas Base.

**Table 1:** Number of samples collected in each campaign from different environments.

Sampling campaign	Sampling date	Code	Ornithogenic soil	Penguin feces	Sea water and sediments	Meltwater streams	Lake water and sediments	Ice from glacier	Microbial mat	Total amount of samples	Total amount of isolates
1	March 2012	M	7	0	3	4	23	4	6	47	102
2	December 2012	D	6	1	3	3	1	0	0	14	23
3	January 2013	E	5	4	9	5	2	0	0	25	39
4	March 2013	FM	2	2	2	4	2	0	0	12	18
5	December 2013	Z	6	0	4	2	0	0	0	12	17
	Total amount of samples		26	7	21	18	28	4	6	110	
	Total amount of isolates		43	7	38	28	67	7	9		199

The last column shows the number of isolates obtained in each campaign. All isolates presented the capacity to grow under denitrifying conditions with nitrate as electron acceptor at 4 °C.

During the sampling temperature was measured. For water samples temperature measured corresponds to the temperature of the sample but for solid samples the ambient temperature was registered. Coordinates of the sampling points were determined using a GPS (Supplementary Table S1).

### Media and Isolation Procedure

In the first sampling campaign, samples were collected, kept at 4°C, transported to Montevideo, Uruguay, and processed in the lab. For the following sampling campaigns, samples were processed in the Artigas Base in Antarctica and then transported to the lab in Montevideo for incubation. Different isolation strategies were applied for the samples collected in March 2012 (first campaign) in order to determine the optimal culture conditions. Depending on the type of sample different media (Table 2) and strategies were used (Supplementary Information).

**Table 2:** Media used to isolate denitrifying bacteria.

Media	Nitrate concentration	Carbon source	Description
PY-N stock solution	10 mM	YE 5 g/l, Peptone 5 g/l	Water with Yeast extract (YE), peptone and potassium nitrate.
Seawater agar	–	Endogenous carbon	1 l of seawater from Collins glacier bay with 13 g agar
R2A-N	5 mM	Several	Commercial R2A broth (Difco) with potassium nitrate
TSB-N	5 mM	Several	Commercial TSB (Difco) with potassium nitrate
BCY-SN	7 mM	Acetate, succinate and ethanol, 10 mM each. YE.	Mineral media (Etchebehere et al., 2001) with potassium nitrate and three defined carbon sources.

All incubations were performed under anaerobic conditions. For the liquid media, the anaerobic atmosphere was obtained by flushing the vials with N_2_ (99.9% Linde, Uruguay). Vials were closed with butyl rubber stoppers and aluminum crimps and sterilized before inoculation. The anaerobic atmosphere for solid media on plates was obtained by using the commercial anaerobic bags (Anaerocult^®^ A mini, Merck, Germany).

All the cultures were incubated at 4°C in a cold chamber until macroscopic evidence of growth was observed. For strain isolation from solid media, colonies with different morphologies were purified by re-streaking a single colony in the same medium used for the isolation under aerobic conditions. The capacity of the isolates to grow in anaerobic conditions using nitrate was confirmed by growth in 1/10 diluted TSB-N media at 4°C under anaerobic conditions. When growth was observed macroscopically, nitrate and nitrite were measured using Nitrate/Nitrite test strips (Quantofix). Only the isolates that presented total consumption of nitrate and nitrite were selected for future analysis.

### Characterization by 16S rRNA Gene Sequencing

Isolated strains were grown in 1/10 diluted TSB-N. In total, 1.5 ml of the culture was centrifuged at 12,000 rpm for 5 min and the pellet resuspended in 10 μl of sterilized ultrapure water. Cells were heat-shock lysed (heated at 100°C for 15 min and incubated in ice for 5 min). 16S rRNA gene was amplified from 1 μl of the lysate. Primers 27F (5′-AGAGTTTGATCCTGGCTCAG-3′) and 1492R (5′-AAGGAGGTGATCCAGCCGCA-3′) were used as previously described (Castelló et al., 2011). The molecular weight of the PCR products were confirmed by agarose gel electrophoresis. The PCR products were purified and the forward strand was sequenced by Capillary Electrophoresis Sequencing using the primer 27F at Macrogen Inc. (Seoul, Korea).^1^ Sequences obtained were processed using Chromas (Technelysium Pty Ltd., version 2.6.5) and trimmed until the average quality of 10 bases exceeds 20. Sequences were further checked manually by observing the sequencing chromatogram to ensure a correct trimming of sequences. In order to classify isolates at genus level, sequences were compared to deposited sequences in the National Center for Biotechnology Information (NCBI)^2^ database using nucleotide-BLAST search and sequences from the EzBioCloud Database.^3^ In Supplementary Table S2, top hit taxon and strain (EzTaxon) and similarity (%) are shown. Sequences were deposited in the GeneBank under the accession numbers: OL843163-OL843361. To further study the diversity of the isolates, a classification in clusters were performed as follows.

### Clustering of the 16S rRNA Sequences Using Phylogenetic Tree

In order to analyze the diversity of strains, we classified strains into clusters according to their 16S rRNA gene sequence. This was performed only as a sequencing clustering method and not to classify sequences taxonomically. For this analysis, four groups of sequences were considered: Group 1: *Pseudomonas* sequences; Group 2: *Janthinobacteria* sequences; Group 3: *Flavobacteria* sequences; and Group 4: All other sequences. Group 1 was the most complicated group as the 16S rRNA gene for several *Pseudomonas* species is highly similar. To obtain reliable clusters we performed a tree including only the longest sequences (above 939 bp). Isolates with sequences shorter than this were not included in the phylogenetic tree but were added individually into the long sequence tree to determine their cluster. This did not disturb the topology of the tree. For Group 2, the tree was also performed with long sequences (971 bp). For shorter sequences same procedure as for group 1 was followed. For *Janthinobacteria*, all sequences grouped in one cluster. For *Flavobacteria* (Group 3), sequences were trimmed to 751 bp and for group 4 to 537 bp.

**Figure 2:**
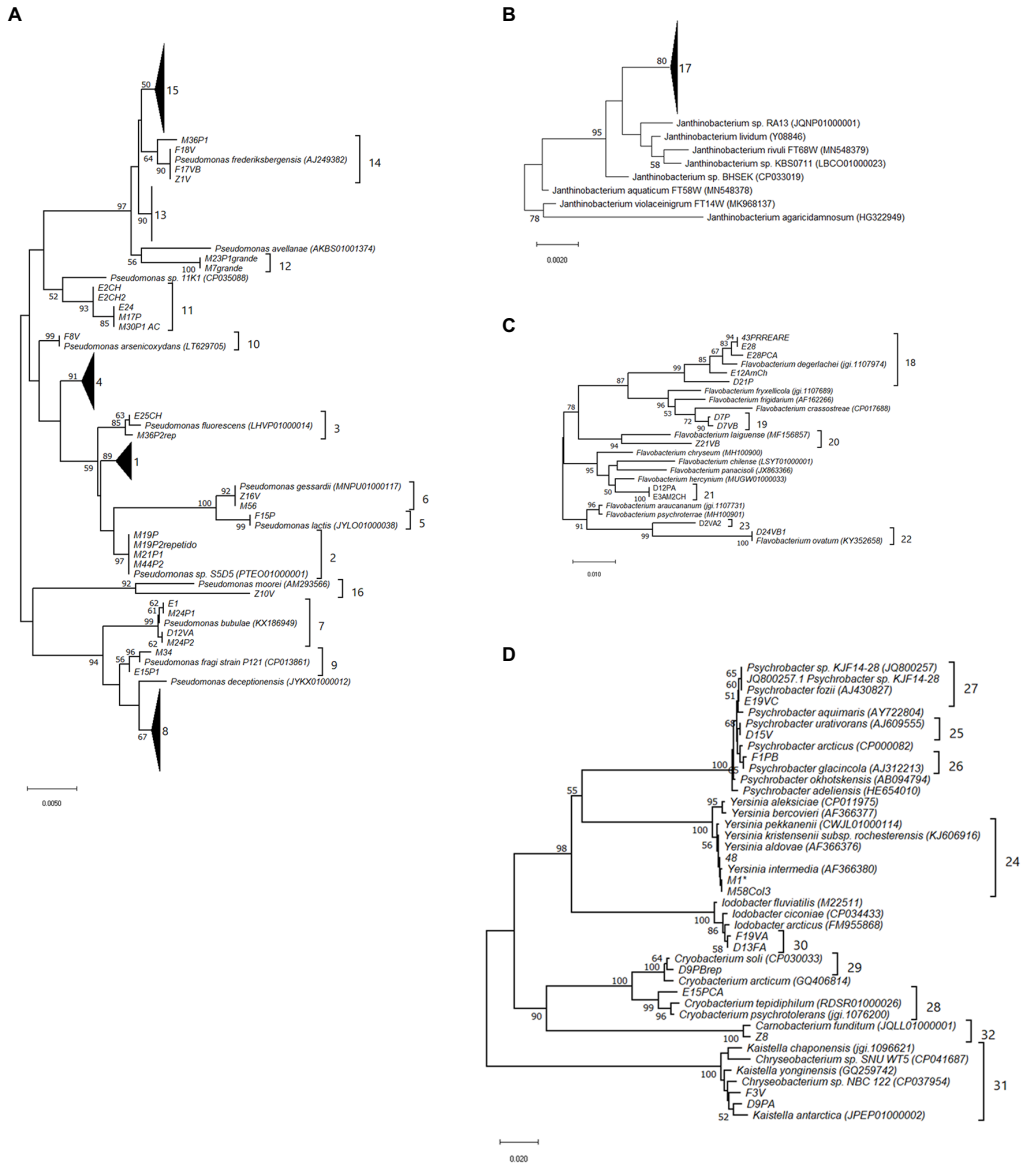
Phylogenetic trees showing the clusters defined for the isolates based on 16S rRNA gene sequences similarity. The evolutionary history was inferred using the Neighbor-Joining method. The percentage of replicate trees in which the associated taxa clustered together in the bootstrap test (1,000 replicates) are shown next to the branches. The tree is drawn to scale, with branch lengths in the same units as those of the evolutionary distances used to infer the phylogenetic tree. The evolutionary distances were computed using the Maximum Composite Likelihood method and are in the units of the number of base substitutions per site. All ambiguous positions were removed for each sequence pair (pairwise deletion option). Evolutionary analyses were conducted in MEGA X [4]. **(A)** *Pseudomonas* sequences, there were a total of 939 positions in the final dataset, **(B)** *Janthinobacterium* sequences. There were a total of 971 positions in the final dataset, **(C)** *Flavobacterium* sequences. There were a total of 751 positions in the final dataset, **(D)** *Psychrobacter*, *Yersinia*, *Cryobacterium*, *Iodobacter*, *Kaistella*, and *Carnobacterium* sequences. There were a total of 537 positions in the final dataset.

Phylogenetic trees were generated using MEGA X (Tamura et al., 2011; Kumar et al., 2018) from 16S rRNA gene sequences from the isolates and from closely related bacteria obtained from NCBI (nucleotide blast using reference RNA sequence database) and EZtaxon. Alignment files for all sequences were generated using the Muscle algorithm (Edgar, 2004) and sequences trimmed to 939 bp, 971 bp, 751 bp, and 537 for groups 1, 2, 3, and 4, respectively). The evolutionary history was inferred by using the Neighbor-Joining method (Saitou and Nei, 1987). The percentage of replicate trees in which the associated taxa clustered together in the bootstrap test (1,000 replicates) are shown next to the branches. The tree is drawn to scale, with branch lengths in the same units as those of the evolutionary distances used to infer the phylogenetic tree. The evolutionary distances were computed using the Maximum Composite Likelihood method and are in the units of the number of base substitutions per site. All ambiguous positions were removed for each sequence pair (pairwise deletion option). In the phylogenetic trees, isolated strains were grouped in 32 different clusters where tree topology, sequence similarity (%) obtained from the distance matrix and sequence similarity to characterized strains from EZ taxon were considered (Supplementary Tables S2**–**S4; Figure 2). Defined sequence branches (bootstrap values higher than 50%, with a reference sequence) with average sequences similarity higher than 97% (generally higher than 99% in most clusters) and consistent results in the EZtaxon database comparison, were considered a cluster. Sequences within the cluster were defined as belonging to the same phylotypes (clusters are shown in bracket next to the branch). The definition of phylotypes is not a taxonomic classification but only a way to classify the sequences in groups with high sequence homology.

### Denitrification Tests Performed by the Acetylene Block Technique

The denitrification capacity at low temperature of isolates from each cluster was confirmed by measuring the accumulation of N_2_O using the acetylene block technique (Mahne and Tiedje, 1995). The acetylene was added to the headspace to inhibit the N_2_O reductase and in order to assess N_2_O accumulation. For this test, firstly, isolates were grown at 4°C in BCY (liquid basal mineral medium; Etchebehere et al., 2001) supplemented with potassium acetate (40 mM) and potassium nitrate (20 mM), under anaerobic conditions (as explained above). Secondly, 1 ml of this culture was transferred into 25 ml vials with 10 ml of the same medium under anaerobic conditions. Immediately after inoculation, acetylene 10% (v/v; 99.9%, Linde, Uruguay) was added to the headspace. The cultures were then incubated at 4°C until growth was observed macroscopically. The accumulation of N_2_O was determined in samples taken from the headspace by GC (GC-2014 Shimadzu) equipped with an electron capture detector and two Porapack Q columns 80/100 6 ft. × ⅛ inch. Total N_2_O content was calculated from the headspace concentration as described by Christensen and Tiedje (1988). Strains that completely reduced nitrate and nitrite, and converted 5%–50% of nitrate to N_2_O were considered weak denitrifiers (+) and conversions above 50% were considered strong denitrifiers (Table 3).

**Table 3:** Phylotype’s characterization indicating their taxonomy, number of isolates, denitrification capacity at 4°C and denitrification genes detected in the closest relative.

	Classification according to EZ taxon	Number of isolates within the phylotype	N_2_O accumulation at 4°C^a^	Nitrite reducatase	Nitrous oxide reductase
1	*Pseudomonas extremaustralis 14-3(T) (AHIP01000073)*	*9*	*+*	*nirK precursor*	*+*
2	*Pseudomonas sp. S5D5 (PTEO01000001)*	*7*	*++*	−	*–*
3	*Pseudomonas fluorescens DSM 50090(T) (LHVP01000014)*	*3*	*+*	*nirS*, *nirK*	*+*
4	*P. fluorescens NCIMB 11764 (CP010945); P. fluorescens PS858 (CABVIR010000015)*	*15*	*+*	*nirS*	*+*
5	*Pseudomonas lactis DSM 29167(T) (JYLO01000038)*	*1*	*+*	*−*	*−*
6	*Pseudomonas gessardii CIP105469 (AF074384)*	*2*	*+*	*−*	*−*
7	*Pseudomonas bubulae TH39(T) (KX186949)*	*12*	*+*	*−*	*−*
8	*Pseudomonas deceptionensis DSM 26521 (JYKX01000012)*	*30*	*++*	*−*	*−*
9	*Pseudomonas fragi P121 (CP013861)*	*5*	*+*	*−*	*−*
10	*Pseudomonas arsenicoxydans VC-1 (FN645213)*	*1*	*++*	*−*	*−*
11	*Pseudomonas sp. 11 K1 (CP035088); Pseudomonas sp. S35 (CP019431)*	*8*	*++*	*nirS*	*+*
12	*Pseudomonas avellanae BPIC 631 (AKBS01001374)*	*6*	*++*	*−*	*−*
13	*Pseudomonas mandelii CIP 105273 (AF058286)*	*15*	*++*	*nirS*	*+*
14	*Pseudomonas frederiksbergensis JAJ28 (AJ249382)*	*5*	*++*	*nirS*	*+*
15	*Pseudomonas sp. In5 (LIRD01000005); Pseudomonas sp. ACM7 (CP024866); Pseudomonas sp. 31–12 (CP029482)*	*22*	*++*	*−*	*−*
16	*Pseudomonas moorei RW10 (AM293566)*	*1*	*+*	*−*	*−*
17	*Janthinobacterium svalbardensis JA-1 DQ355146*	*29*	*++*	*nirK*	*−*
18	*Flavobacterium degerlachei DSM 15718 (jgi.1107974)*	*6*	*++*	*−*	*−*
19	*Flavobacterium frigidarium A2i (AF162266)*	*2*	*+*	*−*	*−*
20	*Flavobacterium laiguense LB2P30(T) (MF156857)*	*1*	*+*	*−*	*−*
21	*Flavobacterium chryseum CCM 8826(T) (MH100900)*	*3*	*−*	*−*	*−*
22	*Flavobacterium ovatum W201E(T) (KY352658)*	*1*	*−*	*−*	*−*
23	*Flavobacterium psychroterrae CCM 8827(T) (MH100901)*	*1*	*−*	*−*	*−*
24	*Yersinia intermedia ATCC 29909 (AF366380)*	*3*	*+*	*−*	*−*
25	*Psychrobacter urativorans DSM 14009 (AJ609555)*	*2*	*−*	*−*	*−*
26	*Psychrobacter glacincola DSM 12194 (AJ312213)*	*1*	*−*	*−*	*−*
27	*Psychrobacter fozii NF23 (AJ430827)*	*1*	*++*	*−*	*−*
28	*Cryobacterium psychrotolerans CGMCC 1.5382(T) jgi.1076200*	*1*	*+*	*−*	*−*
29	*Cryobacterium soli GCJ02(T) CP030033*	*1*	*++*	*−*	*−*
30	*Iodobacter fluviatilis ATCC 33051/M22511*	*2*	*++*	*−*	*−*
31	*Kaistella antarctica LMG 24720(T) JPEP01000002*	*2*	*++*	*−*	*−*
32	*Carnobacterium funditum DSM 5970/JQLL01000001*	*1*	*+*	*−*	*−*

a + indicates N_2_O accumulation between 5% and 50%, ++ indicates N_2_O accumulation between 51% and 100%.

### Search for Denitrification Genes in Public Databases

A bioinformatic approach was used to determine the presence of denitrifying genes in the bacterial species most closely related to the strains isolated in this work. Genes coding for dissimilatory nitrite reductase (*nirS* and *nirK*) and nitrous oxide reductase (*nosZ*) were searched for each taxon. The first step was to create multifasta files to be used as queries; it contained reference sequences covering the diversity spectrum of each gene. The reference gene sequences used to build the multifasta files were selected according to the phylogenetic trees by Graf et al. (2014); (*nirS* and *nirK*) and Jones et al. (2014); (*nosZ*). The nosZ gene sequences were divided into nosZI and nosZII according to Jones et al. (2014), the multifasta files contained 5 and 7 sequences, respectively. The *nirK* gene sequences were divided into two groups according to Graf et al. (2014); group 1 and 2 contained 14 and 10 sequences, respectively. The *nirS* multifasta file contained 8 sequences. The search for *nrf* gene of the DNRA (Dissimilatory Nitrate Reduction to Ammonia) pathway was also performed as N_2_O can also be produced as a byproduct by this pathway. For this, the multifasta file used as query in the search of *nrf*A genes contained 18 sequences from organisms representing 15 different genera (Welsh et al., 2014).

These multifasta files were used as queries in BLAST searches against the nucleotide collection of the NCBI (nr/nt). The searches targeted exclusively the nucleotide sequences annotated as belonging to the taxon most closely related to our isolates according to the 16S rRNA gene sequence analysis, as shown in Table 3. Two different BLAST searches were done for each combination of multifasta file and taxon, one using megablast and one using blastn algorithm in both cases default search parameters were used. Megablast retrieves sequences highly similar to the query while blastn is able to retrieve more dissimilar sequences. All megablast hits were checked and a positive result was considered if the hit sequence was annotated as a denitrifying gene. Blastn resulted in a high number of hits and only those with a hit score higher than 40 were examined; results were considered positive using the same criteria employed for megablast.

## Results

### Isolation and Taxonomic Classification of Potential Denitrifying Bacteria

A total of 199 strains were isolated from 110 samples taken during the five different sampling campaigns (Table 1). All these strains had the capacity to grow under anaerobic conditions at 4°C with nitrate as electron acceptor. The consumption of nitrate and nitrite was verified for all isolates.

The taxonomic classification of the isolates was performed by 16S rRNA gene sequences analysis. Sequences between 450 bp and 1,082 bp were retrieved from all isolates and compared against the database EzBioCloud (Supplementary Table S2). The isolates were classified into 9 different genera with a high predominance of the genus *Pseudomonas*, followed by *Janthinobacterium*, *Flavobacterium*, *Psychrobacter*, and *Yersinia*. Other minor genera detected were *Cryobacterium*, *Iodobacter*, *Kaistella*, and *Carnobacterium* (Table 4).

**Table 4:** Classification of the isolates in the different genera according to the 16S rRNA gene sequence analysis.

Genera	Amount of isolates	Percentage of isolates	Amount of phylotypes
*Pseudomonas*	142	71.4	16
*Janthinobacterium*	29	14.6	1
*Flavobacterium*	14	7	6
*Yersinia*	3	1.5	1
*Psychrobacter*	4	2	3
*Cryobacterium*	2	1	2
*Iodobacter*	2	1	1
*Kaistella*	2	1	1
*Carnobacterium*	1	0.5	1
Total	199	100	32

The number of phylotypes in each genus is shown.

**Figure 3:**
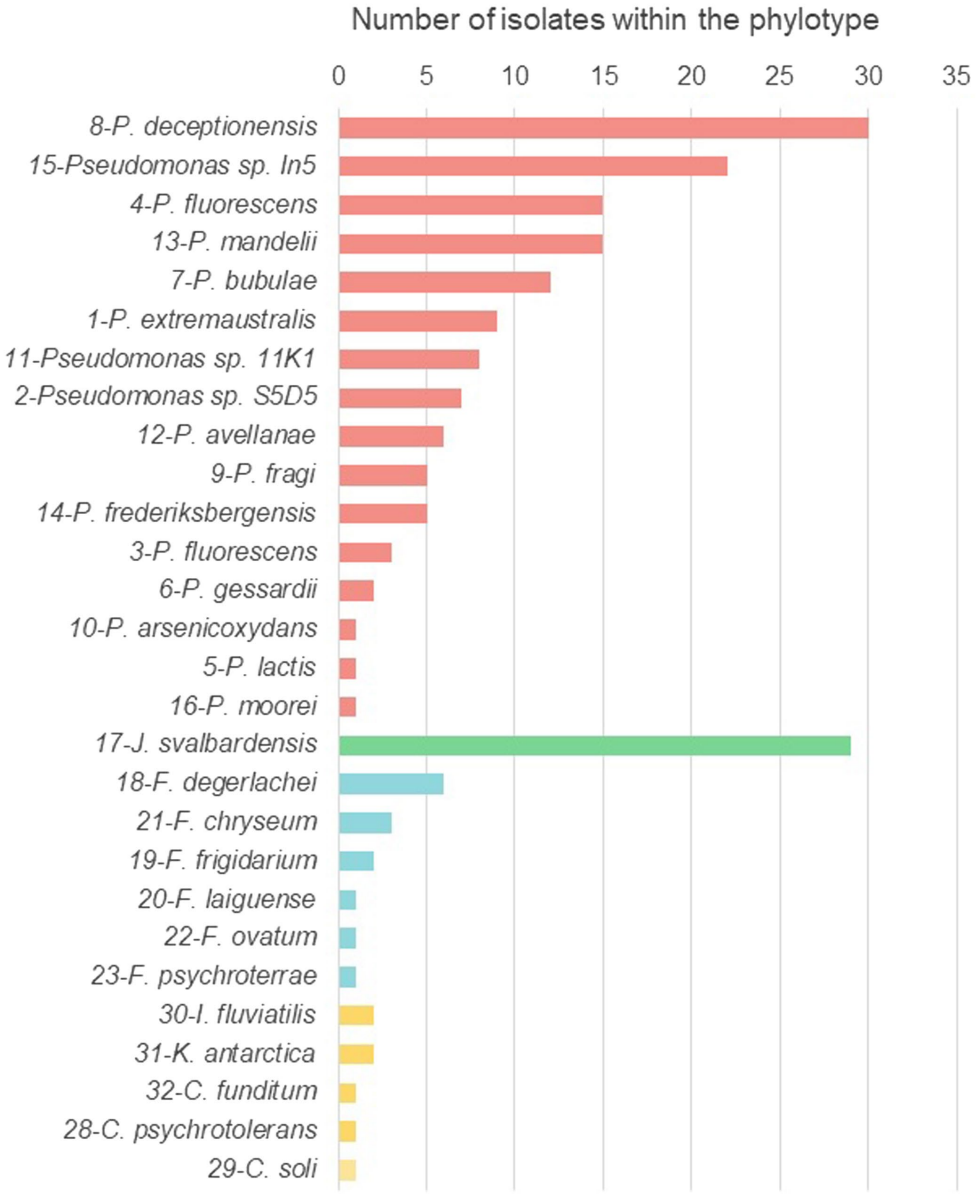
Number of isolates in each phylotype. The phylotype number is indicated next to the classification in the Y-axis. Red: *Pseudomonas*; Green: *Janthinobacterium*; Blue: *Flavobacterium*; Yellow: Other minor genera.

In order to group the isolates according to their 16S rRNA gene sequences homology, phylogenetic trees were built including the sequences from the isolates and their closest relatives retrieved from the databases. Phylotypes were defined as the sequences clustering into the same branch with a bootstrap value higher than 50%. According to this analysis, the isolates were clustered in 32 different phylotypes (Figure 2; Supplementary Table S2). Differences in the amount of phylotypes detected between the two most abundant genera were observed. While *Pseudomonas* isolates clustered in 16 different phylotypes, all *Janthinobacterium* isolates were grouped into one phylotype (Table 4; Figure 2). Out of 32 phylotypes, 29 included more than one isolate and three phylotypes encompass 41% of the isolates, phylotype 8 classified as *Pseudomonas deceptionensis* (30 isolates), phylotype 17 classified as *Janthinobacterium svalbardensis* (29 isolates), and phylotype 15 classified as *Pseudomonas* sp. In5 (22 isolates; Figure 3; Supplementary Table S2).

### Distribution of Phylotypes in the Different Environments and Sampling Campaigns

**Figure 4:**
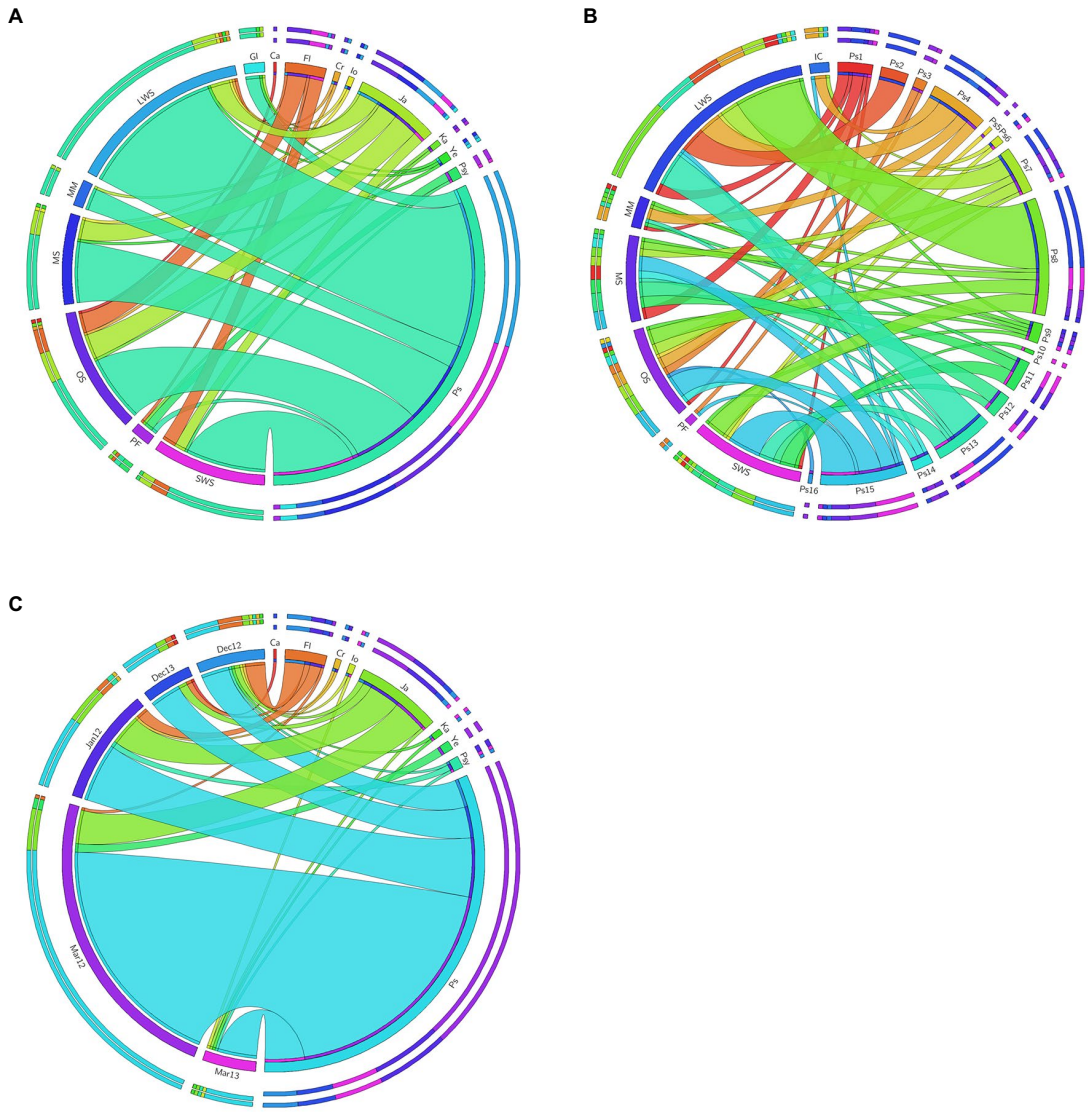
The chart links the genera **(A)** and the *Pseudomonas* phylotypes **(B)** with the sampled ecosystem and, the genera with the sampling date **(C)**. Width of the linking bands are proportional to the number of isolates. The fraction of the circumference that the phylotype or sample type covers are proportional to the number of isolates obtained for each phylotype or from each sample type, respectively. Sample types are labeled with two uppercase letters, and phylotypes are labeled with “Ps” followed by the respective number. Sample types (in counter clockwise order): GI, ice from glacial; LWS, lake water and sediment; MM, microbial mat; MS, meltwater stream; OS, ornithogenic soil; PF, penguin feces; SWS, sea water and sediment. Genera (in clockwise order): Ca, *Carnobacterium*; Fl, *Flavobacterium*; Cr, *Cryobacterium*; Io, *Iodobacter*; Ja, *Janthinobacterium*; Ka, *Kaistella*; Ye, *Yersinia*; Psy, *Psycrhobacter*; Ps, *Pseudomonas*.

The predominant phylotypes were detected in samples collected from most environments, indicating that they are ubiquitous in this Antarctic region. In Figure 4, we show that each genus is linked to more than one ecosystem. And in more detail, Figure 4B shows that the same occurs for each phylotype within *Pseudomonas*. We did not find one specific phylotype associate to a specific environment.

One of the aspects we wanted to study was if there was an ecological succession of denitrifying population throughout the deicing period. For this, samples were taken during 2 years, in several periods of the deicing season (beginning of the deicing: December; middle: January; and end of the deicing season: March). Especially because in December most of the lake water was frozen but mostly thawed by March. Moreover, in the end of the deicing season, many meltwater streams and microbial mats emerged. Again, isolates from the most abundant genera were found in all the sampling campaigns including the ones from the beginning of the deicing period (Figure 4C).

### N_2_O Accumulation Test at 4°C

To confirm the denitrifying capacity of the isolates at cold temperature, strains from the different phylotypes were selected and tested for N_2_O accumulation. It is important to consider that similarities between the 16S rRNA gene sequence of strains within clusters is different and therefore the strains might not be representative of the diversity within a cluster. From the 32 isolates tested, 27 were positive indicating that a high proportion of the isolates have the capacity to denitrify in psychrophilic conditions. A total of 14 positive isolates converted more than 50% of the nitrate to N_2_O (Table 3). From all genera detected we found at least one isolate within each genera, with the capacity to accumulate N_2_O at 4°C. All isolated tested within Pseudomonas presented the capacity to denitrify at 4°C. While within *Flavobacterium*, three out of six strains tested were able to denitrify at 4°C (Table 3). These results show that the strategy used to isolate denitrifying strains at low temperature was successful.

### Search for Denitrifying Genes in the Databases

According to the classification of the 16S rRNA gene sequences for each phylotype, the denitrifying organisms isolated in this work, were closely related to strains from cultured microorganisms. Regarding their reported denitrifying capacity, we found that most of the species related to our isolates have not been described as capable of denitrification. Only within *Pseudomonas* we found that *Pseudomonas mandelii* (Verhille et al., 1999) and *Pseudomonas frederiksbergensis* (Andersen et al., 2000) have been described as denitrifiers. Frequently, denitrification is not tested in the description of new species so we decided to perform a search for genes encoding denitrification pathway enzymes in the databases.

For this, a bioinformatic approach was performed to look for the genes encoding denitrification pathway enzymes (*nirS*, *nirK*, and *nosZ*) in the closest relative taxa. The results showed that, in most cases, the denitrifying genes were not found for these microorganisms (Table 3). Only 7 of the 27 phylotypes positive for the N_2_O accumulation test were related to taxa in which denitrification genes were detected. Interestingly, the phylotypes 7, 8, and 15, which have more than 10 isolates each, are not related to described denitrifiers and no genes related to the denitrifying pathways were found in the database (Table 3).

Within the three predominant genera detected, the 16 phylotypes related to *Pseudomonas* spp. tested for N_2_O accumulation gave positive results, but only 6 were related to species or taxa with reported denitrifying genes. The *Janthinobacterium* phylotype (17) classified as *Janthinobacterium svalbardensis* JA-1 DQ355146, which has a *nirK* gene. For the phylotypes classified within the genus *Flavobacterium*, the N_2_O accumulation test gave variable results but none of the related species presented denitrifying genes in the bioinformatic analysis.

Regarding nitrous oxide reductase genes (*nosZ*), only six phylotypes, all within *Pseudomonas*, were closely related to a nosZ-encoding bacteria. Five of these six phylotypes that has nosZ, also presented nitrite nitrite reductase genes.

None of the four phylotypes that presented negative N_2_O tests were related to species with denitrifying genes.

Regarding the search for the *nrf*A gene of the DNRA pathway, only *Yersinia aleksiciae* DSM 14987(T)p resented the sequence of *nrf*A gene in the genome. The *Yersinia* isolates (48, M1* and M58col3) are not closely related to this species but to *Yersinia intermedia* (Figure 2; Supplementary Tables S2, S4).

## Discussion

### Key Denitrifying Populations in Antarctica

In this work, denitrifying bacteria were isolated from several samples from Antarctica, from December to March. We observed predominance of the genera *Flavobacterium*, *Pseudomonas*, and *Janthinobacterium*. The taxonomic classification was performed to genus level as to accurately classify the isolates, genomic sequencing of each strain would be necessary.

The description of the genus *Flavobacterium* dates back to 1923 (Bernardet and Bowman, 2006), but the taxonomy of its members has been revised extensively in recent years. Moreover, many new species have been described in the last few decades. Of the 7 species identified in our survey, 6 were originally isolated from the Antarctica (Humphry et al., 2001; Van Trappen et al., 2004; Kim et al., 2014; Kralova et al., 2018; Ren et al., 2018) while the remaining (*F. laiguense*) was isolated from the Tibetan Plateau (Yang et al., 2019). Denitrification genes have not been described for any of these species so far but nitrate reduction has been observed for some of them. However, most isolates have shown to be negative for nitrate reduction and unable of anaerobic growth according to the publications of the sp. nov. description.

Regarding the genus reports of denitrfying nitrite reductase genes of *Flavobacterium* species are scarce. For instance, *Flavobacterium* sp. BH12.12 was positive for *nirS* amplification in a survey from estuary sediment in the United Kingdom (Nogales et al., 2002). Other *Flavobacterium* species have been isolated as denitrifying microorganisms but their denitrifying genes have not been described so far, such is the case of *Flavobacterium denitrificans* (Horn et al., 2005) and *F. aquicola* (Hatayama et al., 2016). At the genome level, *Flavobacterium columnare* is the only one with identified *nirK* genes (Tekedar et al., 2017). These precedents, along with our results, suggest that species of *Flavobacterium* may host yet undescribed denitrifying genes.

Most isolates that were classified as *Pseudomonas* spp. were closely related to *P. deceptionensis*. This specie was originally isolated from the Deception Island in the Antarctica (Carrión et al., 2011) which can be indicative of the preference of this taxon for cold environments and a strong biogeographic factor affecting its distribution. However, it is surprising the number of isolates that we obtained as closely related to this specie considering that there are no reports of denitrification activity or genes for it. Likewise, *Pseudomonas extremaustralis* isolated from the Antarctic Peninsula was negative for denitrification (López et al., 2009). However, denitrification genes are present in the genome as indicated by the whole genome sequencing results (Raiger Iustman et al., 2015).

A total of 12 strains of *P. bubulae* were isolated in our study. This specie was recently described and was isolated from raw refrigerated beef (Lick et al., 2020) insinuating a psychrophilic preference of the specie no denitrifying evidence were reported. Less surprising is the isolation of 15 strains of *P. mandelii*. This bacterial specie was demonstrated to be able to denitrify (Verhille et al., 1999) and denitrifying genes has been already described (Saleh-Lakha et al., 2009). Moreover, a psychrotolerant strain of *P. mandelii* was originally isolated from marine sediment taken from the Fildes Bay at King George Island (Vásquez-Ponce et al., 2017).

The first reported strain of *Janthinobacterium svalbardensis* was isolated from ice glacial in the arctic (Ambrožič Avguštin et al., 2013). But, the ability to denitrify was not tested for that strain. However, *J. svalbardensis* F19 was shown to be able to perform aerobic denitrification and its *nirS* gene was obtained by PCR (Chen et al., 2019). Also, *nirS* and *nirK* genes, but not *nosZ*, are present in the genome of *J. svalbardensis* PAMC 27463 (Cho et al., 2017). It may therefore be not surprising to easily isolate strains related to this taxon when looking for denitrifiers in cold environments.

In order to better understand the role of these denitrifying isolates further studies are needed regarding their phylogeny and biochemistry. For example, sequencing their genome and more physiological characteristics like optimal temperature growth, capacity to perform aerobic denitrification, etc. would give relevant information to deepen our knowledge on the relevance of these genera in Antarctic environments. Moreover, even though DNRA (Dissimilatory Nitrate Reduction to Ammonia) genes are less abundant than denitrification genes in Antarctic soils (Ramírez-Fernández et al., 2021) and that the gene *nrf*A was not present in the genomes closely related to the isolates, it would be interesting to study the capacity of the strains to perform DNRA.

### Denitrification in Antarctic Environments

Nitrogen cycle has been previously reported in Antarctic environments (Cowan, 2014), but the denitrification pathway has not been extensively studied. Then, it still remains unknown how Antarctic extreme conditions impact denitrifiers, and, if there are particular groups of denitrifiers selected in this cold environment. Denitrification can be studied either by cultivation and isolation using specific conditions for denitrifiers or by detecting previously described genes or proteins directly in environmental samples (Table 5). We only found three works focusing on isolation of denitrifiers from samples taken from Antarctica (Ward and Priscu, 1997; Powell et al., 2006; Lanzarotti et al., 2011). In the first work, denitrifiers were isolated from lake water samples but their identity was not described. In the work performed by Powell et al. (2006), the authors were focused on isolation of denitrifiers from hydrocarbon contaminated soils and they complemented using specific qPCR and DGGE to track denitrifiers. The authors described a high proportion of *Pseudomonas* within the isolates. These results are in accordance with our results which also showed the predominance of *Pseudomonas* within the denitrifying isolates. More recently, Lanzarotti et al. (2011), isolated a bacteria classified as *Bizionia argentinensis and* denitrifying genes (nirS, norB, and nosZ) were found in its genome. The authors indicate that this capacity has been rarely reported for *Flavobacteriaceae* members (Jones et al., 2008; Lanzarotti et al., 2011). In our work, we isolated 14 denitrifying strains within the *Flavobacteriaceae* family, specifically *Flavobacteria*. Probably, this capacity is underestimated in this family and more research is needed to reveal the denitrifying capacity in cold environments.

**Table 5:** Studies performed on denitrifying bacteria in samples from diverse ecosystems in Antarctica.

Antactic environment	Method	Genera detected	Reference
Lake water	Isolation	Not reported	Ward and Priscu, 1997
Hydrocarbon contaminated soils	Isolation, qPCR (nir and nos), DGGE (nosZ)	*Pseudomonas*	Powell et al., 2006
Soil	Microarray with funcional genes for N_2_ cycle and qPCR	Not reported	Yergeau et al., 2007
Antarctic surface seawater	Isolation and genome sequencing	*Bizionia argentinensis* (nirS, norB, nosZ)	Lanzarotti et al., 2011
Soil	narG, nirK, nirS, nosZ qPCR	not reported	Jung et al., 2011
Soil	narG, nirS, nirK, norB, nosZ qPCR	not reported	Han et al., 2013
Microbial mats	nirS, nirK, and nosZ DGGE	*Octadecabacter*, *Rubrivibax*, *Paracoccus*, *Rhodopseudomonas, Azospirillum, Rhodoferax, Pseudomonas*	Alcántara-Hernández et al., 2014
Soil	nirS and nirK amplicon sequencing	*Pseudomonas* (nirS), *Mesorhizobium* (nirK), *Nitrosospira* (nirK), *Desulfosarcina* (nirK)	Dai et al., 2020
Soil	Metagenome assembled genomes	*Pandoraea thiooxydans* (nirS, norB, nosZ), *Oblitimonas alkalihpila* (nirS, norB, nosZ), *Pseudomonas stutzeri* (nirS, norB, nosZ), *Rhizobacter gummiphilus* (nirS, norB), Nitrospira lacus (nirK, norB), *Rhodoferax ferrireducens* (nirS, nosZ), *Dokdonella koreensis* (nirS, nosZ), Rhodoferax saldenbachensis (nirS), *Alicyclobacillus acidocaldarius* (nirS), *Truepera radiovictrix* (nirS), Wenzhouxiangella marina (nirS), *Pseudoxanthomonas suwonensis* (nirS), Flavisolibacter tropicus (nirK), Pusillimonas sp. (nirK), Methylovorus glucosetrophus (norB, nosZ), *Thiobacillus denitrificans* (norB, nosZ), Dyella jiangningensis (norB), Gemmatinmonas phototrophica (norB), *Variovorax paradoxus* (norB), *Akkermansia muciniphila* (norB), Lysobacter capsicii (norB), *Gemmatimonas aurantica* (norB), *Haliscomenobacter hydrossis* (norB), *Rhodanobacter denitrificans* (nosZ), *Janthinoacterium* sp. (nosZ), *Sulfuriferula* sp. (nosZ)	Ramírez-Fernández et al., 2021
Soil, lake water and sediments, sea water and sediments, ice, meltwater, microbial mats, penguin feces, soil.	Isolation and denitrification test at 4°C.	*Pseudomonas*, *Janthinobacterium*, *Flavobacterium*, *Psychrobacter*, *Yersinia*, *Cryobacterium, Iodobacter*, *Kaistella*, and *Carnobacterium*	This work

Denitrifying bacteria have also been detected using culture independent methods. Using functional gene detection by PCR, the presence of denitrifiers in samples taken from the Antarctic has been described, but by this technique, it was not possible to determine the identity of the denitrifiers (Powell et al., 2006; Yergeau et al., 2007; Jung et al., 2011). Another PCR-based technique used is DGGE with functional gene specific primers. For example, Alcántara-Hernández et al. (2014), detected the presence of many denitrifying genes in soil samples. Some of the genes detected were closely related to genes from the genera *Octadecabacter*, *Rubrivibax*, *Paracoccus*, *Rhodopseudomonas*, *Azospirillum*, *Rhodoferax*, and *Pseudomonas* while many of the genes observed could not be classified. From all these genera we detected *Pseudomonas* in all the samples analyzed confirming the ubiquity of this bacteria. Another technique based on functional gene detection but with no PCR-bias was used by Yergeau et al. (2007). Using functional gene microarrays, the authors analyzed samples taken from a transect from the Falkland Islands to the Antarctic continent. They also verified the presence of many genes from the denitrifying pathway along the transect, with a positive correlation between their abundance and the temperature. In this work the identity of denitrifiers was not described.

Using a metagenomics approach in samples taken from animal impacted soils, and recovering microbial assembled genomes (MAGs) from the metagenomes, Ramírez-Fernández et al. (2021), described several MAGs harboring denitrifying genes in their genomes. This result also indicated the ubiquity of this pathway in the microorganisms from the Antarctic. From the high diversity of genera observed, two of them (*Pseudomonas* and *Janthinobacterium*) were also found in our study. Regarding the relative abundances of the isolates, as different isolation strategies were used and we were not always able to sample the same ecosystems during the sampling campaigns, the results would not be comparable.

Then, our literature survey showed that denitrification is ubiquitous in the Antarctic ecosystems, confirming our finding as we were able to isolate denitrifiers from all analyzed samples. Moreover, we were able to isolate denitrifiers from samples that has never been studied before as ice, meltwater, sea sediment and water, and penguin faeces. However, it has to be taken into account that the climate of maritime Antarctica is different from continental Antarctica and therefore, the denitrifying diversity might also change and denitrification should be further studied to completely understand their role in cold environments.

### Which Comes First, Chicken or the Egg? Isolation or Detection of Known Functional Genes to Track Denitrifiers

Different methods have been used to study denitrifying bacteria in the Antarctic. Each method has its bias; isolation only detects bacteria capable of growing in the lab, functional gene based analysis, only recovers genes that have been previously described. Therefore, it is not easy to define an experimental strategy to detect microorganisms with unknown denitrifying pathways. Using metagenomics approach, we can recover genomes from a sample without isolating, which allows to discover new taxa. But, the denitrifying gene search within the MAGs are performed by similarity with known sequences, so only genes which has already been described will be found.

Our work contributed to the knowledge of denitrifiers in the Antarctic ecosystem but, we were not able to describe the genes using a bioinformatics approach. Sequencing their genomes would allow us to identify known denitrifying genes. If the bacteria have new genes, experiments of gene transcription or protein expression in different conditions (with and without nitrate) in order to identify candidate genes or proteins will be necessary. After this, in order to verify the role of the gene, mutagenesis experiments would be needed.

Functional metagenomics is a culture independent approach used to identify new genes and could be an alternative to recover new denitrifying genes from environmental samples.

So, even though molecular methods advance there is still a need to isolate strains and perform physiological studies in order to unravel the role of denitrifying strains and their denitrification pathways.

## Conclusion

Denitrification capacity was detected in all ecosystems analyzed and during the whole deicing season. Denitrification is ubiquitous in the different ecosystems present in the maritime Antarctica. We verified that most isolates were able to perform denitrification at low temperature (4°C) linking function with taxa in an unequivocal way. This capacity has not been described for most of the bacteria related to our isolates and in many of them denitrifying genes were not present in the databases suggesting the presence of new genes in extreme environments. More research is needed to unravel this knowledge gap. This work is a contribution to further understanding the role of denitrifying bacteria in Antarctica.

## Data Availability Statement

The datasets presented in this study can be found in online repositories. The names of the repository/repositories and accession number(s) can be found at: National Center for Biotechnology Information (NCBI) BioProject database under accession numbers OL843163-OL843361.

## Author Contributions

AC performed experiments, analyzed results, and wrote the manuscript. GA analyzed results and wrote the manuscript. PB-W, LB, and JW performed sampling and experiments. LF, SS, and ST performed experiments. CE planned the experiments, performed sampling and experiments, analyzed results, and wrote the manuscript. All authors contributed to the article and approved the submitted version.

## Funding

This work was supported by a project grant funded by the Antarctic Institute from Uruguay.

## Conflict of Interest

The authors declare that the research was conducted in the absence of any commercial or financial relationships that could be construed as a potential conflict of interest.

## Publisher’s Note

All claims expressed in this article are solely those of the authors and do not necessarily represent those of their affiliated organizations, or those of the publisher, the editors and the reviewers. Any product that may be evaluated in this article, or claim that may be made by its manufacturer, is not guaranteed or endorsed by the publisher.

## Footnotes

^1^ https://dna.macrogen.com/main.do

^2^ http://ncbi.nlm.nih.gov

^3^ https://www.ezbiocloud.net/
