# Supplementary material for: Ubiquity and Diversity of Cold Adapted Denitrifying Bacteria Isolated From Diverse Antarctic Ecosystems
Source: Front Microbiol. 2022 Jul 18;13:827228. doi: 10.3389/fmicb.2022.827228 (PMC9339992; doi:10.3389/fmicb.2022.827228)
Supplement: Supplementary file 5 [file Table_5.DOCX]

**Supplementary information**

**Methods**

**Isolation strategies used for the different samples:**

a) Seawater samples

Strategy 1- Samples were enriched by mixing 20 mL of the water sample in a 60 mL sterile vial with sterile PY-N stock solution. Final concentration in the enrichment were: potassium nitrate 1 mM, yeast extract 0.5 g/L and peptone 0.5 g/L. Bacteria were isolated from vials showing growth by streaking on a plates with the same media.

Strategy 2- Direct isolation was performed by streaking the sample on seawater agar plates (Table 2).

Strategy 3- Samples (1 L) were filtered using 0.22 micron sterile filters (millipore) and filter was placed on a seawater agar plate (Table 2).

Strategy 4- Enrichment was performed by adding samples (1mL) to vials with 10 mL R2A-N (broth) liquid medium (Table 2). Bacteria were isolated from vials showing growth by streaking on a plate with the same medium.

Strategy 5- Enrichment was performed by adding 1 mL of the sample to 25mL vials with 10 mL of 1/10 TSB-N (Table 2). Bacteria were isolated from vials showing growth by streaking on a plate with the same medium.

b) Lake water samples: For lake water samples strategies 1 and 5 explained above were used and the following strategies were added:

Strategy 6- 20 mL of water sample was added to a vial with 60 mL of BCY-SN mineral medium (Etchebehere et al., 2001) (Table 2). Bacteria were isolated from vials with macroscopic growth by streaking on a plate with the same medium.

Strategy 7- Bacteria were directly isolated by streaking the sample on plates with BCY-SN medium with 13g/l of agar.

c) Ice and meltwater samples: For ice and meltwater samples strategies 1 and 5 explained above were used and the following strategies were added:

8- Samples (0.5 mL) were spread on plates with BCY-SN medium supplemented with agar (Difco, 13 g/L).

d) Ornithogenic soil, penguin feces, sediments and microbial mat samples: For solid samples strategies 5 and 6 were used with the following modifications: A first step to suspend the samples was performed by mixing samples with 5 mL of NaCl (9 g/L). Then, two enrichment and isolation procedures were used with two media, BCY-SN (strategy 6) and TSBN (strategy 5). For strategy 6, 2.5 mL of the suspension were inoculated into 25 mL vials containing 10 mL of BCY-SN and continued as described above. For strategy 5, 1 mL of the suspension was inoculated in TSB-N, and continued as described above.

For the following sampling campaigns, the isolation strategy 5 was applied to all samples. For lake and sea water samples one additional strategy was applied: 1 mL of water sample was filtered using a 0.22 μm sterile filter (Millipore) and the filter was placed on a plate with 1/10 diluted TSB-N with 13 g/L of agar.

**Table S1**-Complete data of the samples including: sampling campaign, location, environment description, Temperature and amount of isolates obtained from each sample.

**Table S2**- Complete data of each isolate including: samples location and environment, 16S rRNA gene sequences length, accession number, EzBioCloud sequence comparison result, phylotype. ^a^: Strains used as representative for the Acetylen block test.

**Table S3-** Table shows averages sequence similarities (%) between isolates for all clusters. Average sequence similarities were calculated from the distance matrices obtained using MEGA X for each cluster.

**Table S4.** Average sequence similarities matrices for all sequences and clusters calculated from the distance matrices obtained using MEGA X.
